# The prognostic value of [^123^I]-vascular endothelial growth factor ([^123^I]-VEGF) in glioma

**DOI:** 10.1007/s00259-018-4088-y

**Published:** 2018-07-30

**Authors:** Eva Rainer, Hao Wang, Tatjana Traub-Weidinger, Georg Widhalm, Barbara Fueger, Jingling Chang, Zhaohui Zhu, Christine Marosi, Alexander Haug, Marcus Hacker, Shuren Li

**Affiliations:** 1 Division of Nuclear Medicine, Department of Biomedical Imaging and Image-guided Therapy, Medical University of Vienna, Vienna, Austria; 2 Department of Nuclear Medicine, Peking Union Medical College (PUMC) Hospital, Chinese Academy of Medical Science and PUMC, Beijing, China; 3 Beijing Key Laboratory of Molecular Targeted Diagnosis and Therapy in Nuclear Medicine, Beijing, 100730 China; 4 Department of Neuro-Surgery, Medical University of Vienna, Vienna, Austria; 5 Department of Biomedical Imaging and Image-guided Therapy, Medical University of Vienna, Vienna, Austria; 6 Department of Neurology, Dongzhimen Hospital, Beijing University of Chinese Medicine, No. 5 Haiyuncang, Beijing, 100700 Dongcheng District China; 7 TCM EncePhaloPathy Treatment Key Laboratory of Beijing University of Chinese Medicine, Beijing, China; 8 Department of Internal Medicine I, Division of Oncology, Medical University of Vienna, Vienna, Austria

**Keywords:** VEGF, Angiogenesis, Scintigraphy, Brain, Glioma, Prognosis

## Abstract

**Purpose:**

Recent studies have shown that tumor vascular endothelial cells and various tumor cells overexpress receptors for vascular endothelial growth factor (VEGF). The aim of this study was to investigate the prognostic value of [^123^I]-VEGF scintigraphy in patients with histologically verified brain tumors.

**Methods:**

23 consecutive patients (9 women and 14 men aged 30–83 years, mean age 56.6 ± 14.4 years) with histopathologically-verified primary brain tumors were included in the study. All patients had undergone [^123^I]-VEGF scintigraphy. SPECT examinations of brain were performed 30 min and 18 h after injection. Additional [^11^C]-methionine PET ([^11^C]-MET PET) was performed in eight of the 23 patients. Both [^123^I]-VEGF and [^11^C]-MET PET were evaluated visually and semiquantitatively by tumor-to-normal brain uptake *ratio (T/N* ratio). Thresholds of the T/N ratio were evaluated by analysis of receiver operating characteristics (ROC). Overall survival (OS) was estimated using the Kaplan-Meier method.

**Results:**

World Health Organization (WHO) grade IV glioma lesions showed [^123^I]-VEGF uptake 18 h after the injection, whereas other brain tumors of grade II or III showed negative results. There was no significant difference in the tumor size between VEGF positive and VEGF negative tumors. Patients with [^123^I]-VEGF T/N ratio threshold <1.32 showed significantly longer survival than patients with T/N ratio ≥ 1.32 (2680 days vs 295 days; *P* < 0.05). In the subgroup of 16 grade IV glioma patients, significant OS differences were found using a T/N ratio of 1.75 as threshold (T/N ratio < 1.75: 720 days; T/*N* ≥ 1.75: 183 days; *P* < 0.05). Significant difference (*P* < 0.05) was also found in [^11^C]-MET PET T/N ratios between the grade IV glioma (mean T/N ratio: 3.71) and the grade II or III glioma (mean T/N ratio: 1.74).

**Conclusion:**

Our results suggest that [^123^I]-VEGF scintigraphy may be useful for visualization of tumor angiogenesis. In addition, [^123^I]-VEGF may provide relevant prognostic information in patients with glioma.

## Introduction

Angiogenesis is essential for the growth of many tumors and metastases. Recent studies have demonstrated that human glioblastoma (World Health Organization (WHO) grade IV glioma) overexpresses proangiogenic factors, including vascular endothelial growth factor (VEGF) [1, 2]. VEGF binds to VEGF receptor 1 (VEGFR1, Fms-like-tyrosine kinase (Flt-1)) and VEGFR2 (kinase domain region (KDR), Flk-1), which may activate endothelial cell proliferation, migration and survival [3, 4]. It has been shown that VEGF receptors (VEGFRs) are over-expressed in both grade IV glioma vasculature and grade IV glioma cells [5]. Previous studies demonstrated that VEGF and its corresponding receptor Flk-1 are significant prognostic factors for overall survival in patients with glioma [6, 7]. Therefore, in vivo imaging of VEGFR expression may be useful for tumor diagnosis and monitoring of response to anti-VEGF/VEGFR therapy [4, 8]. For imaging of VEGFR expression, VEGF has been labeled with various radioisotopes [9–12]. However, to our best knowledge, all these studies were experimental and results of clinical studies are not available. A recent imaging study assessing tumor uptake of a radiolabeled anti-VEGF antibody, such as the monoclonal antibody bevacizumab in glioma, showed unsatisfactory results with significant inter- and intra-patient heterogeneity [13]. The antiangiogenic treatment with bevacizumab has been a major research focus for patients with recurrent and newly diagnosed malignant gliomas in recent years [14]. However, results of interventional trials with bevacizumab have been disappointing [14, 15]. Therefore, it is very important to identify and validate reliable biomarkers for optimization of antiangiogenic therapy for individual patients; VEGFRs could be valuable imaging targets in the course of antiangiogenic therapy. Up to now, no data are available on the imaging of human brain tumors using the radiolabeled VEGF. Based on our previous studies [16–18], in this prospective study we evaluated for the first time the prognostic role of [^123^I] - vascular endothelial growth factor ([^123^I]-VEGF) in patients with histologically confirmed brain tumors.

## Methods

### Patients

Between March 2005 and April 2008, 23 patients with a newly diagnosed primary brain tumor or with a recurrent brain tumor (9 women and 14 men aged 30–83 years, mean age 56.6 ± 14.4 years) were included in this prospective study (Table 1). Patients with metastatic brain tumors were not considered for participation in the study. Inclusion criteria were initial radiological diagnosis of a primary intra-axial brain tumor or suspected recurrence or progression after treatment of a histologically confirmed intra-axial primary brain tumor. Tumor diagnosis was established by the local neuropathology team according to the WHO classification of tumors of the central nervous system within our study period [19, 20]. This prospective trial was approved by the ethics committee of the Medical University of Vienna. The written informed consents were obtained from all patients. None of the patients had signs of clinically apparent inflammation or infection at the time of the study.

**Table 1:** Characteristics and results of patients with brain tumor

Patient no.	Gender	Age (years)	Final histology and WHO grade	Therapy before VEGF scan	Lantency to VEGF scan (months)	Therapy after VEGF scan	Tumor size (cm)	OS (days)	VEGF SPECT ^a^ Visual T/N	MET PET Visual SUV ^b^; T/N
1	M	45	Oligodendroglioma II	no therapy	n.a.	OP, RX, CX	4,5	3231	Neg 0.90	Pos 3.17; 2.58
2	F	76	GB IV	no therapy	n.a.	OP	5	6	Pos 1.80	n.p.
3	M	83	GB IV	no therapy	n.a.	OP, RX	4	52	Pos 1.76	n.p.
4	M	68	GB IV	no therapy	n.a.	RX	7	91	Pos 1.73	n.p.
5	F	30	Astrocytoma II	no therapy	n.a.	Epilepsy therapy	5,3	4325	Neg 0.87	Pos 2.52; 1.42
6	F	69	B cell Lymphoma	no therapy	n.a.	CX	2,5	371	Neg 1.18	n.p.
7	M	64	GB IV	no therapy	n.a.	OP, RX, CX, Bevacizumab + Irinotecan	3	661	Pos 1.75	n.p.
8	F	58	GB IV	no therapy	n.a.	RX, CX	6	152	Pos 2.24	n.p.
9	M	53	GB IV	no therapy	n.a.	OP, RX, CX	1,7	399	Pos 1.90	Pos 3.20; 2.83
10	M	66	GB IV	no therapy	n.a.	OP, RX, CX	5	53	Pos 1.78	Pos 3.77; 2.60
11	M	60	GB IV	no therapy	n.a.	OP, RX	5	218	Pos 2.80	n.p.
12	M	59	GB IV	Partial resection	2	RX, CX	5,5	57	Pos 1.78	n.p.
13	F	46	GB IV	RX	0.25	OP, RX, CX, Imatinib, Bevacizumab	3	1162	Neg 0.90	n.p.
14	M	38	Anaplatic ganglioglioma III	partial resection, RX	20	no therapy	3,6	739	Neg 1.00	Pos 3.51; 1.56
15	M	39	GB IV	RX, CX	36	Bevacizumab + Doxorubicin	3,9	1353	Pos 1.45	n.p.
16	F	57	Oligodendroglioma II	OP, RX, CX	24	CX, Imatinib, Thalidomide	7	1963	Neg 1.03	Pos 10.41; 1.47
17	M	75	GB IV	OP, CX	6	OP, RX	1,7	403	Pos 1.56	n.p.
18	M	53	GB IV	Stereotactic biopsy	1	OP, RX, CX	5	329	Pos 1.59	Pos 6.91; 5.71
19	fF	55	GB IV	OP, RX	2	RX	2,4	55	Pos 1.84	n.p.
20	F	67	GB IV	no therapy	n.a.	RX, CX	1,5	158	Neg 1.07	n.p.
21	M	41	Anaplastic oligoastrocytoma III	CX, RX	5	OP	2	5021	Neg 1.06	Pos 4.20; 1.68
22	F	37	Haemangiopericytoma II	partial resection, RX	60	Bevacizumab	3,5	1214	Neg 0.85	n.p.
23	M	62	GB IV	partial resection, RX, CX	3	RX, CX	4,5	577	Pos 1.65	n.p.

GB: glioblastoma, n.a.: not applicable, RX: radiotherapy, CX: Chemotherapy, OP: surgery; Pos: positive scan; Neg: negative scan; n.p.: not performed.^a^Results of VEGF SPECT at 18 h post injection ^b^The maximal standardized uptake values (SUV) for the tumor

### VEGF synthesis and SPECT imaging

Recombinant human VEGF_165_ (rhVEGF_165_) (PromoCell GmbH, Heidelberg, Germany) was labelled with ^123^I by the method described previously [17] with a radiochemical purity higher than 97%.

[^123^I]-VEGF was administered slowly as a single intravenous bolus injection over 2 min in an average dose of 140±16 MBq [≤130 pmol (≤5 μg) VEGF per patient]. In order to determine the hemodynamic effects of VEGF, blood pressure and heart rate were monitored during tracer application and scintigraphy. The patients received 200 mg sodium perchlorate three times daily over 3 days for thyroid blockage.

All acquisitions were obtained with double-headed and large field of view camera (Millennium VG with Hawkeye; GE Medical Systems, Milwaukee, Wisc., USA) employing medium-energy high-resolution collimators [21]. Dynamic acquisition of the head was initiated immediately after intravenous administration of [^123^I]-VEGF and carried out until 30 min post injection. SPECT (159 keV 20% window, MEHR Collimator, 128 × 128 Matrix; 30 min) was performed at 30 min and 18 h post injection. Whole body images were acquired in anterior and posterior views at 1 h post injection.

One patient with grade IV glioma was examined by [^123^I]-VEGF before and 1 week after the radiation therapy, respectively.

### SPECT data analysis

Raw imaging data were reconstructed using the Butterworth-filtered back projection algorithm, generating tomographic views of the brain in the three planes (transverse, coronal, and sagittal). Radiotracer accumulation in the brain tumor was firstly assessed visually. For the calculation of the tumor-to-normal brain uptake (the T/N ratio), a region of interest (ROI) was manually drawn around the lesion on the transverse slice—with close reference to the corresponding MRI/CT slice—and a reference region was drawn on the contralateral tumor-free brain side. The maximum count rate in the lesion ROI and the mean count rate in the reference ROI were used for calculation the T/N ratio according to a previous publication [22].

Scintigraphic results from the [^123^I]-VEGF scans were directly compared with consecutive histopathological analyses, which were used as gold standard. MRI and CT were used for lesion allocation as available.

### [^11^C]-methionine positron emission tomography ([^11^C]-MET PET)

Eight patients had additional [^11^C]-MET PET examinations. Other patients had no [^11^C]-MET PET either due to immediate start of therapy (*n* = 10) or rapid progression of the disease (*n* = 5). The intervals between [^123^I]-VEGF scan and [^11^C]-MET PET were between 5 and 26 days (12 ± 7 days (mean ± SD); median 9 days). The [^11^C]-MET was produced by the method described previously by Mitterhauser et al. [23] with a radiochemical purity higher than 97%. For PET acquisition, a dedicated full-ring GE Advance PET scanner (General Electric Medical Systems) was used for all patients (field of view: 14.875 cm, 35 slices per PET examination with a slice thickness of 4.25 mm). An average dose of 750 ± 66 MBq MET was intravenously injected 20 min prior to the PET start. Data acquisition was undertaken for another 15 min in 3D mode. A transmission scan for attenuation correction was performed afterwards. Subsequently, the image reconstruction was done by filtered back projection using a Hanning filter with a cutoff value of 6.2 mm and a 128 × 128 matrix.

### [^11^C]-MET PET data analysis

All reconstructed PET data were transferred from the GE system and evaluated by experienced nuclear medicine physicians using a dedicated Hermes software (Gold 3 Hermes Hybrid Viewer, Hermes Medical Solutions). For visual assessment, brain tumors were analyzed for hypo-, iso-, or hypermetabolism of MET, as described previously [24]. Hypo- or isometabolism of brain tumors was summarized as MET-negative brain tumors. For semiquantitative analysis, cuboid VOIs (20 mm × 20 mm × 20 mm) were manually generated containing the highest tracer uptake of the tumor and for background estimation in the contralateral brain. For the calculation of the T/N ratio, the maximal standardized uptake value (SUV) of the tumor was divided by the mean SUV of the background VOI. If no tracer uptake was seen, VOIs were drawn based on MRI.

### Statistical analysis

Comparisons between groups were performed with t-tests and Chi-square tests, where appropriate. Overall survival (OS) was defined as the time of the histopathological diagnosis to the date of death or the date of last follow-up. OS was calculated by the Kaplan-Meier method. Univariate analyses were performed by means of Cox regression. Correlation studies were evaluated by linear regression analysis and the Pearson or Spearman correlation analysis, where appropriate. The optimal threshold values for VEGF uptake T/N ratios in relation to survival were determined from receiver operating characteristic (ROC) curves. A *P* value <0.05 was considered statistically significant. All statistical analyses were performed using Sigma Plot version 11.0 (Systat Software Inc., California, USA).

## Results

### [^123^I]-VEGF uptake in brain tumors

The paper-electrophoresis and TCA precipitation analysis of the plasma samples 30 min after [^123^I]-VEGF administration showed that over 85% (*n* = 3) of the activity was present as unchanged [^123^I]-VEGF. After intravenous injection of [^123^I]-VEGF, patients showed no clinical adverse reaction, and no side effects were noted.

**Fig. 1:**
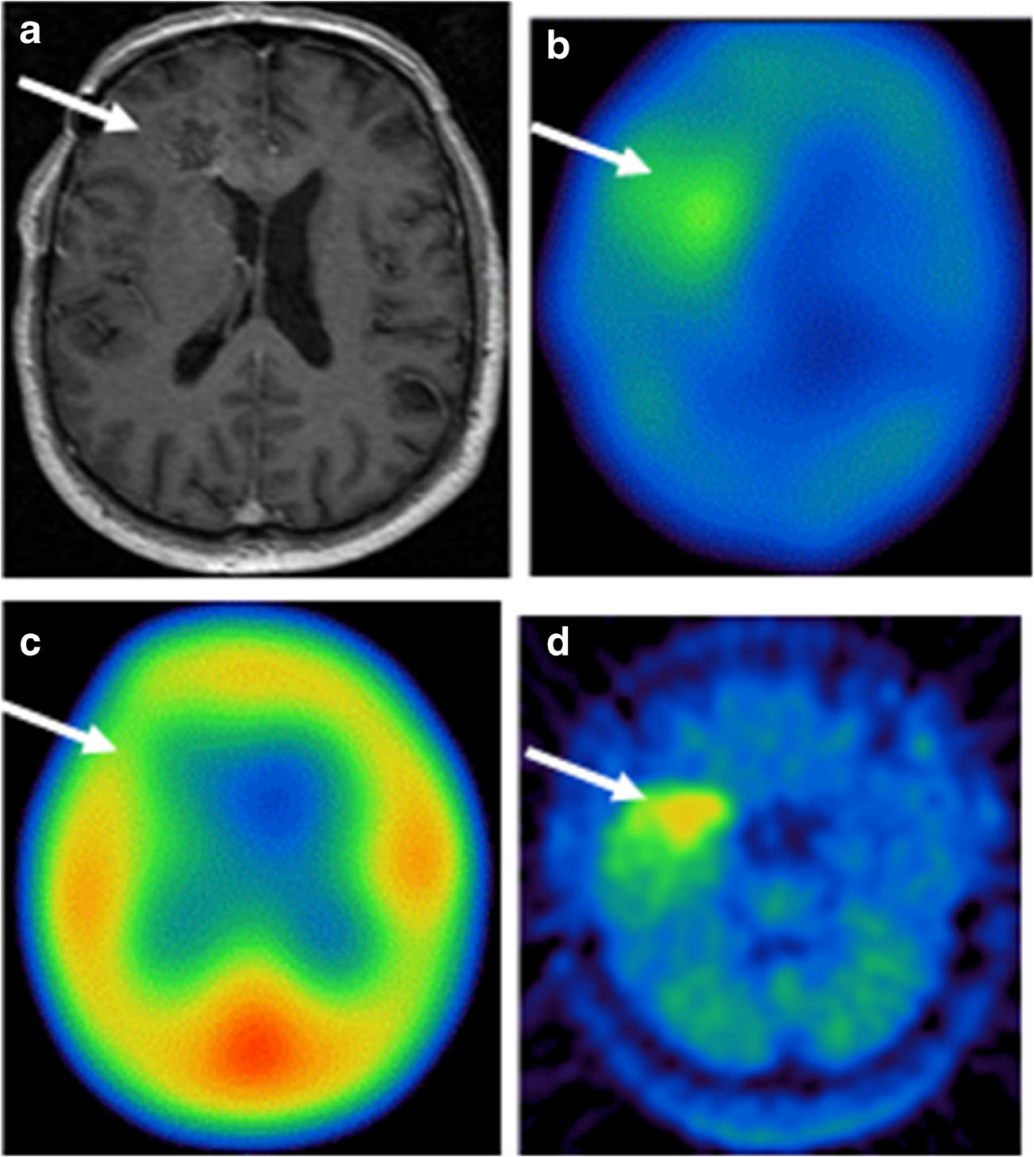
A 66-year-old man with grade IV glioma in the right frontal lobe. Axial T1-weighted MRI showed the lesion in the right frontal lobe with enhancement (**a**). The tumor lesion was positive in [^123^I]-VEGF SPECT imaging 18 h after injection of [^123^I]-VEGF (**b**), and no significant [^123^I]-VEGF accumulation was demonstrated 30 min after the [^123^I]-VEGF application (**c**). The lesion was positive in [^11^C]-MET PET (**d**). White arrows indicate the tumor region

No significant [^123^I]-VEGF accumulation was demonstrated 30 min after [^123^I]-VEGF application in all patients (Fig. 1). In the [^123^I]-VEGF scans 18 h after injection, 14/16 patients with WHO grade IV glioma showed VEGF uptake (Fig. 1), while 2/16 patients remained negative. One patient had a tumor diameter of 1.5 cm; the other patient had received radiation therapy 1 week prior to the [^123^I]-VEGF scan. Other brain tumors (2 oligodendrogliomas of grade II, 1 anaplastic oligoastrocytoma of grade III, 1 astrocytoma of grade II, 1 anaplastic ganglioglioma of grade III, 1 haemangiopericytoma of grade II, and 1 diffuse large B cell lymphoma) showed negative [^123^I]-VEGF results (Table 1).

The mean diameter of tumors in VEGF scan negative patients was 3.6 ± 1.8 cm (range 1.5–7.0 cm), while in VEGF scan positive patients the mean diameter of tumors was 4.3 ± 1.6 cm (range 1.7–7.0 cm). There was no significant difference in the mean tumor size between VEGF positive tumors and VEGF negative tumors (*P* = 0.333).

There was a significant correlation between the [^123^I]-VEGF scintigraphy results and classification of brain tumors (*P* < 0.05). All positive tumors were grade IV glioma, while all brain tumors of grade II and III had negative VEGF scan results.

**Fig. 2:**
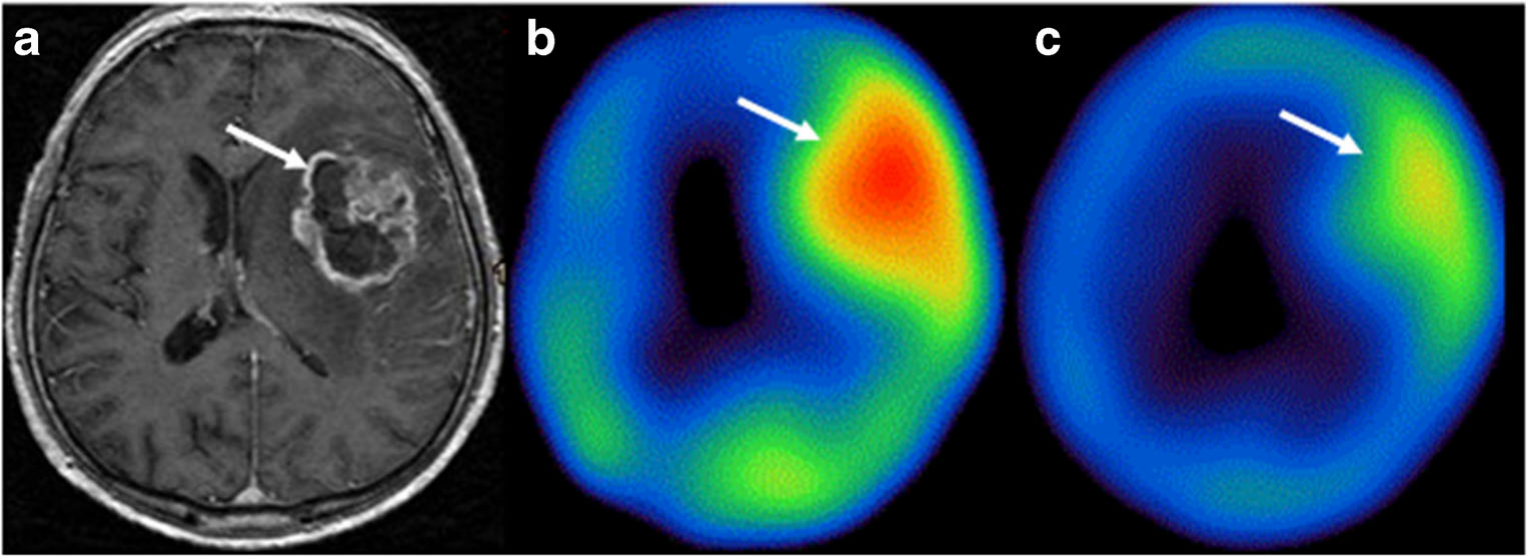
A 60-year-old man with grade IV glioma. Axial T1-weighted MRI showed the lesion in the left frontal-temporal lobe with enhancement (**a**). The tumor lesion was highly positive in [^123^I]-VEGF SPECT imaging (the T/N ratio = 2.80) before the radiation therapy (**b**). Reduced [^123^I]-VEGF accumulation in the tumor region (the T/N ratio = 1.78) was found after the radiation therapy (**c**). *White* *arrows* indicate the tumor region

In one patient with grade IV glioma, [^123^I]-VEGF scan was performed before and a week after radiation therapy, respectively. The [^123^I]-VEGF scan results showed a reduced [^123^I]-VEGF uptake in the tumor region after the radiation therapy. The T/N ratio before radiation therapy was 2.80, whereas the T/N ratio after radiation therapy was 1.78 (Fig. 2).

### [^123^I]-VEGF uptake and OS

Overall survival (OS) was registered in the 23 patients with brain tumors during a follow-up period of mean 32 months. Kaplan-Meier analysis revealed a significantly shortened OS in patients with positive VEGF scan (*P* = 0.009).

Comparison of the mean survival days between patients with positive VEGF scan results and patients with negative VEGF scan results showed that there were significant differences (*P* = 0.003) in mean OS between the patients with positive VEGF scans (315 ± 365 days) and the patients with negative VEGF scans (2020 ± 1768 days) (Table 1).

**Fig. 3:**
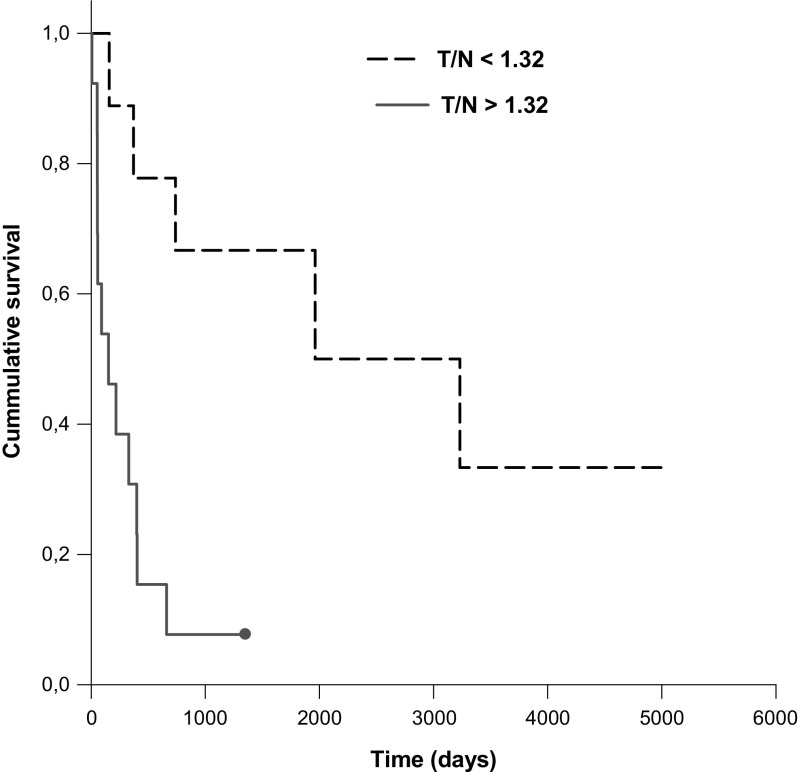
[^123^I]-VEGF SPECT T/N ratio and survival (*P* = 0.002) in patients with brain tumors

For separating grade IV glioma from other brain tumors a T/N ratio threshold of 1.32 in [^123^I]-VEGF SPECT at 18 h post injection was identified by ROC analysis (area under the cure (AUC): 0.951, sensitivity 88%, specificity 100%). Significant OS difference was shown using the T/N ratio of 1.32 (T/N ratio < 1.32: 2680 days; T/N ratio > 1.32: 295 days, *P* = 0.002) (Fig.3). 14/16 grade IV gliomas had a T/N ratio of >1.32. Only two grade IV gliomas had T/N ratio of <1.32. All other brain tumors had a T/N ratio of <1.26 in [^123^I]-VEGF SPECT (Table 1).

**Fig. 4:**
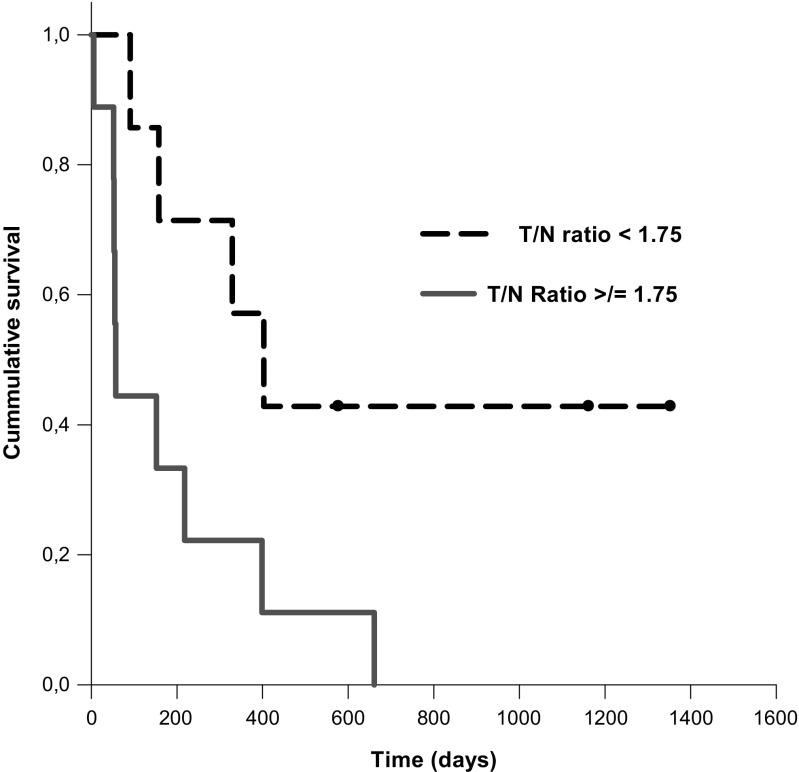
[^123^I]-VEGF SPECT T/N ratio and survival (*P <* 0.05) in patients with grade IV glioma

For estimation of [^123^I]-VEGF as a prognostic marker in the group of grade IV glioma, a T/N ratio threshold of 1.75 in [^123^I]-VEGF SPECT at 18 h post injection was used based on the ROC analysis. Significant OS difference was observed in grade IV glioma patients with [^123^I]-VEGF SPECT T/N ratio < 1.75 (720 days) versus ≥1.75 (183 days; *P* < 0.05; Fig. 4).

### Comparison of [^123^I]-VEGF SPECT with [^11^C]-MET PET

All eight patients, who had both [^123^I]-VEGF SPECT and [^11^C]-MET PET, were positive in [^11^C]-MET PET. Three patients with grade IV glioma were also positive in [^123^I]-VEGF SPECT, while the other five patients with grade II or III glioma (one patient with astrocytoma of grade II, one patient with anaplastic oligoastrocytoma of grade III and one patient with anaplastic ganglioglioma of grade III as well as two patients with oligodendroglioma of grade II) had negative [^123^I]-VEGF SPECT.

There was no significant difference (*P* > 0.05) in the SUV values in [^11^C]-MET PET between the grade IV glioma and the grade II or III glioma. However, a significant difference (*P* < 0.05) was found for T/N ratios in [^11^C]-MET PET between the grade IV glioma (mean T/N ratio: 3.71) and the grade II or III glioma (mean T/N ratio: 1.74).

The T/N ratios of [^11^C]-MET PET and [^123^I]-VEGF SPECT at 18 h post injection showed no significant correlation (γ = 0.625, *P* > 0.05).

## Discussion

In the present study, the direct binding of [^123^I]-VEGF to human brain tumors in vivo was shown for the first time. Interestingly, our study showed that only WHO grade IV glioma was positive in [^123^I]-VEGF, and negative [^123^I]-VEGF scintigraphy results were found in the less malignant tumors, including grade II and III gliomas and a B cell lymphoma. A previous in vitro study [25] supports our results. Chan et al. [25] have demonstrated that overexpression of both VEGFR1 and VEGFR2 was found in the tumor vasculature of grade IV glioma; whereas a weak or no expression of VEGFRs was found in the astrocytomas of grade II, anaplastic astrocytomas, and oligodendroglioma tumors. Therefore, the low number or absence of VEGF receptors may explain negative scintigraphic results in patients with grade II or III glioma or B cell lymphoma. Thus, [^123^I]-VEGF scintigraphy might indicate specific biological characteristics (activity or angiogenetic state) of brain tumors, which might have implications for both differential diagnosis and biological treatment of brain lesions.

In this study, two patients with grade IV glioma had negative [^123^I]-VEGF results. One patient had received radiation therapy 1 week prior to the [^123^I]-VEGF scan, which might have influenced the VEGF receptor status of this specific tumor. This was additionally proven by another patient with grade IV glioma showing a strong [^123^I]-VEGF uptake before radiation therapy. His post-therapeutic [^123^I]-VEGF scan also showed a reduced [^123^I]-VEGF uptake in the tumor region after radiation therapy. It is too early to make valid conclusions based on these two cases; however, the results appear to have implications for evaluation of therapy response.

The other patient had a tumor with only 1.5 cm diameter, which could be attributed to the limited spatial resolution of our gamma camera. In general, there was no significant difference in mean tumor size between patients with positive and negative [^123^I]-VEGF scan results.

The blood–brain barrier (BBB) is disrupted in grade IV glioma, resulting in the characteristic cerebral edema and contrast enhancement on MRI. Mechanistically, the disrupted BBB may facilitate the effective delivery of [^123^I]-VEGF to the intracranial glioma tumor. However, to our surprise, no increased [^123^I]-VEGF uptake in grade IV glioma was found at 30 min after intravenous injection of [^123^I]-VEGF. This indicates that accumulation of VEGF in the grade IV glioma is not a simple, passive diffusion due to disruption of the BBB. The accumulation of [^123^I]-VEGF in grade IV glioma in the SPECT images obtained 18 h post injection is most likely due to the active uptake of [^123^I]-VEGF through its receptors into grade IV glioma. It has been demonstrated that the neovascularization of grade IV glioma may be different compared to other tumors, reflecting the complex nature of grade IV glioma neovascularization [26] and suggesting the uptake of [^123^I] VEGF through VEGFRs into the endothelial cells of grade IV glioma as a relatively slow process. Anyway, the exact mechanism underlying the breakdown of the BBB in grade IV glioma is still unknown.

In the present prospective study, we explored the ability of [^123^I]-VEGF to assess the prognosis of patients with brain tumor. Our results demonstrated that the OS in patients with a positive VEGF scan was significantly shorter than in patients with a negative VEGF scan. This was strengthened by our quantitative evaluation, where patients with higher T/N ratio had significantly shorter OS as compared to patients with lower T/N ratio, suggesting poor prognosis of patients with high [^123^I]-VEGF uptake. No significant correlation of T/N ratios between MET PET and [^123^I]-VEGF was demonstrated in eight patients who had both [^123^I]-VEGF SPECT and [^11^C]-MET PET. [^11^C]-MET PET is an established clinical method for the evaluation of brain tumors and was found positive in gliomas of different grades (II, III and IV). Furthermore, it was recently published that [^11^C]-MET PET has a significant prognostic impact in treatment-naïve gliomas [24]. Despite a modest number of patients examined with [^11^C]-MET PET, the results of our present study were consistent with previous publications [24, 27].

Interestingly, two patients with grade II or III glioma had negative [^123^I]-VEGF SPECT but positive [^11^C]-MET PET imaging. Both patients are still alive, suggesting superior characteristics for [^123^I]-VEGF in outcome prediction.

The effect of VEGF on tumor growth was not investigated in the present study. It has been reported that intratumoral administration of exogenous VEGF in a murine fibrosarcoma model did not significantly change tumor weight and size as compared to saline controls [28]. In our studies, the concentration of injected VEGF is far below pharmacologically relevant doses and, thus, very unlikely to promote tumor growth. Even therapeutic clinical trials of VEGF delivery at pharmacologic doses in peripheral vascular or coronary disease failed to significantly increase angiogenesis [29].

The in vivo stability of radiolabeled tracers is always a concern, such as those based on iodine radioisotopes. Analysis of the chemical status of plasma radioactivity in the samples of the first 30 min after injection mainly shows VEGF-bound radioiodine in the form of the original [^123^I]-VEGF. Previous studies [30, 31] have shown that the in vivo stability of [^123^I]-VEGF or [^125^I]-VEGF did not significantly influence tumor uptake of the tracer, although there was high radioiodine levels in the thyroid and stomach. Similar findings were also observed in our previous studies using [^123^I]-VEGF [17, 18].

The endogenous isoforms of VEGF may compete with [^123^I]-VEGF, which can potentially influence tracer uptake in the tumor and makes quantitative correlation of VEGFR expression with tracer uptake more difficult. Nonetheless, the intact VEGFR binding affinity of [^123^I]-VEGF and the fact that the endogenous VEGF concentration is far from saturating the VEGFR resulted in [^123^I]-VEGF uptake in the grade IV glioma.

While in other commonly used nuclear medical techniques, as for example [^18^F]-FDG PET, differentiation between normal tissue and pathologic lesions is difficult due to the high glucose metabolism of normal brain tissues, we observed no substantial [^123^I]-VEGF uptake by normal brain tissue. This characteristic of [^123^I]-VEGF biodistribution may be an apparent advantage for the characterization of brain tumors as highly malignant.

There are some limitations of the present study. Firstly, a limited number of patients with grade 2 or 3 glioma were  included and furthermore metastatic brain tumors were not included in the present study. Previous studies [5–7] have demonstrated increased (tumor) angiogenesis in grade IV glioma in vitro, exploring the value of [^123^I]-VEGF for imaging the (tumor) angiogenesis of grade IV glioma would  be very interesting; therefore, more patients with verified or suspect grade IV glioma were included in this prospective pilot study. Secondly, we have performed the SPECT examinations only at 30 min and 18 h post injection. The design of this prospective clinical trial was made based on our previous studies of different tumors [17, 18] and we expected a similar distribution. In the present study, significant accumulation of [^123^I]-VEGF was found only in SPECT at 18 h post injection but not in SPECT at 30 min post injection. We speculate that the uptake of the [^123^I]-VEGF in brain tumors may be slower than that in the other different tumors such as primary pancreatic adenocarcinomas and their liver metastases. Thirdly, [^11^C]-MET PET was not examined for all patients due to start of therapy or rapid progression of the disease, which significantly restricts the estimation of [^11^C]-MET PET as a prognostic marker in the gliomas and limits the comparability between [^11^C]-MET PET and [^123^I]-VEGF.

## Conclusion

[^123^I]-VEGF scintigraphy may be useful for the visualization of the tumor angiogenesis and may have a significant prognostic impact in the patients with glioma.
